# Ecological Niche Modelling of the *Bacillus anthracis *A1.a sub-lineage in Kazakhstan

**DOI:** 10.1186/1472-6785-11-32

**Published:** 2011-12-12

**Authors:** Jocelyn Mullins, Larissa Lukhnova, Alim Aikimbayev, Yerlan Pazilov, Matthew Van Ert, Jason K Blackburn

**Affiliations:** 1 Department of Geography, University of Florida, Gainesville, FL, USA; 2 Spatial Epidemiology and Ecology Research Laboratory, Emerging Pathogens Institute, University of Florida, Gainesville, FL, USA; 3 Kazakh Science Centre for Quarantine and Zoonotic Diseases, Ministry of Health of the Republic of Kazakhstan, Almaty, Kazakhstan; 4 Scientific and Practical Centre of Sanitary and Epidemiological Expertise and Monitoring, Ministry of Health of the Republic of Kazakhstan, Almaty, Kazakhstan

## Abstract

**Background:**

*Bacillus anthracis*, the causative agent of anthrax, is a globally distributed zoonotic pathogen that continues to be a veterinary and human health problem in Central Asia. We used a database of anthrax outbreak locations in Kazakhstan and a subset of genotyped isolates to model the geographic distribution and ecological associations of *B. anthracis *in Kazakhstan. The aims of the study were to test the influence of soil variables on a previous ecological niche based prediction of *B. anthracis *in Kazakhstan and to determine if a single sub-lineage of *B. anthracis *occupies a unique ecological niche.

**Results:**

The addition of soil variables to the previously developed ecological niche model did not appreciably alter the limits of the predicted geographic or ecological distribution of *B. anthracis *in Kazakhstan. The A1.a experiment predicted the sub-lineage to be present over a larger geographic area than did the outbreak based experiment containing multiple lineages. Within the geographic area predicted to be suitable for *B. anthracis *by all ten best subset models, the A1.a sub-lineage was associated with a wider range of ecological tolerances than the outbreak-soil experiment. Analysis of rule types showed that logit rules predominate in the outbreak-soil experiment and range rules in the A1.a sub-lineage experiment. Random sub-setting of locality points suggests that models of *B. anthracis *distribution may be sensitive to sample size.

**Conclusions:**

Our analysis supports careful consideration of the taxonomic resolution of data used to create ecological niche models. Further investigations into the environmental affinities of individual lineages and sub-lineages of *B. anthracis *will be useful in understanding the ecology of the disease at large and small scales. With model based predictions serving as approximations of disease risk, these efforts will improve the efficacy of public health interventions for anthrax prevention and control.

## Background

Anthrax is a disease of wildlife, livestock and humans that remains a public health problem throughout the world. *Bacillus anthracis*, the causative agent of anthrax, is a soil-borne, spore-forming bacterium which persists in soil for long periods of time under appropriate conditions [1]. Certain soil parameters, including pH, organic content and calcium, may be associated with spore survival [1-5]. Anthrax outbreaks among livestock and wildlife result from exposure to these spores and are possibly influenced by climatic and physiological events [6,7]. In endemic areas, human cases of anthrax primarily result from contact with infected livestock during slaughter or butchering [8,9] and control of livestock disease through vaccination and active surveillance of livestock and wildlife is essential for preventing human disease [10]. However, widespread active surveillance is costly and vaccination of every animal is not feasible. It is far more practical to focus these efforts on areas of high risk. To identify these it is necessary to improve our understanding of the ecology of *B. anthracis *through which animal infection occurs.

The ecology of a pathogen such as *B. anthracis *can be explored using similar tools as those used for species distribution modelling and conservation planning. For example, ecological niche modelling (ENM) has been used to predict the potential ecological and geographic distribution of pathogens based on outbreak locations [10-14], presence of disease vectors [15-17] and disease reservoirs [18]. The ecological niche of a pathogen, as for other types of species, is conceptualized as the N-dimensional hypervolume of ecological parameters within which the species can be maintained without immigration [19,20]. Various approaches to ENM identify non-random associations between a species' locality data and environmental parameters. Ecological niche modelling experiments of *B. anthracis *are particularly useful considering the potential associations between spore survival and ecological conditions [1,5]. Results can be used as a proxy for disease risk and integrated into focused surveillance strategies for wildlife and livestock in endemic areas and into vaccination strategies that target at risk herds before and during outbreak events [10].

Recently, studies of disease ecology have combined molecular genotyping techniques and ecological niche modelling to provide evidence that genetic lineages of a pathogen can have different environmental associations and potential geographic distributions [12,21]. In general *B. anthracis *has relatively limited global diversity. However, multiple locus variable number tandem repeat analysis (MLVA) systems for *B. anthracis *can differentiate strains into distinct lineages and sub-lineages [22-24]. Analyses of a global collection of *B. anthracis *isolates suggests that the A lineage is globally distributed, while other lineages (B and C) are geographically restricted. These findings may be explained by adaptive differences, some of which carry fitness costs that limit abundance and distribution of certain lineages or sub-lineages [3,24]. The ecological niche of *B. anthracis *has been modelled in the United States and Kazakhstan using locations of reported outbreaks [10,11,25,26]. A stated limitation of these experiments was that the outbreak data potentially included multiple strains of *B. anthracis *[10,11]. If lineages of *B. anthracis *do exhibit niche specialization and unique geographic distributions, then it is plausible that current outbreak based ecological niche models are biased toward a dominant strain in a particular landscape. It would then follow that single lineage models may better predict presence of the pathogen at local scales and increase the value of public health measures [10].

Kazakhstan is situated in Central Asia, a region with some of the highest reported human anthrax incidence and mortality rates in the world [27,28]. The majority of human anthrax cases in Kazakhstan are related to exposure to infected livestock or handling of products derived from infected livestock [9]. In rural areas of Kazakhstan, veterinary care and surveillance programs are limited by the country's large land mass and widely distributed rural populations. Vaccination of livestock occurs mainly in response to detected outbreaks. In countries such as Kazakhstan, prioritizing areas for vaccination and surveillance are necessary for disease control. Our group recently created a multi-variate ecological niche model to characterize the broad environmental conditions that support *B. anthracis *across Kazakhstan [11,26]. In a parallel effort, Aikimbayev et al. used an eight marker MLVA typing system (MLVA-8) to describe the diversity of *B. anthracis *within Kazakhstan from 88 archival strains [22,29].

In this study, we first expanded on the previously published outbreak based modelling experiment by adding four soil variables (pH, calcium levels, organic content and baseline water saturation) to the original set of environmental variables. Despite literature suggesting a strong relationship between soil characteristics such as high calcium levels and alkaline pH and spore persistence [1,4,5], the influence of available soil variables on *B. anthracis *ENM predictions has not been comparatively examined [10]. We next used these twelve environmental variables and the collection of MLVA-8 genotyped samples to create an A1.a sub-lineage specific ecological niche model for Kazakhstan.

## Results

### Accuracy Metrics

Ecological niche modelling was performed using the Genetic Algorithm for Rule-Set Prediction (GARP). Four experiments were run (outbreak-soil, A1.a sub-lineage, small southern outbreak and large southern outbreak) and are summarized in Table 1. All modelling processes reached convergence of accuracy (0.01) prior to reaching the maximum iteration setting (= 1,000). The outbreak-soil model had an Area Under the Curve (AUC) of 0.7188 and was significantly different from a random model. Total omission of the outbreak-soil model was 2.6% and average omission was 9.9%, indicating that 97.4% of the testing points were predicted by at least one best subset model and 89.1% were predicted by all models. The AUC of the A1.a sub-lineage model was 0.6964 and was also significantly different from a random model and had a total and average omission of 0 and 13.1, respectively. Both the large and small outbreak models had AUCs significantly different than random. Accuracy metrics for all models are shown in Table 2.

**Table 1:** Summary of experiments

Experiment	External Data Split (%Training/%Testing)	Area used for Model Building	Locality Data
Outbreak-Soil	85/15	All Kazakhstan	All spatially unique livestock outbreaks
A1.a Sub-lineage	80/20	Southern Polygon	Spatially unique A1.a isolates in southern polygon
Small Southern Outbreak	85/20	Southern Polygon	Random sub-set of spatially unique livestock outbreaks in southern polygon
Large Southern Outbreak	80/15	Southern Polygon	All spatially unique livestock outbreaks in southern polygon

**Table 2:** Sample sizes and accuracy metrics for GARP model building and evaluation Table 3

	Model			
**Metric**	**Outbreak-Soil**	**A1.a sub-lineage**	**Large Southern Outbreak**	**Small Southern Outbreak**

N to build models*	218	26	113	26
N to test models	39	13	145	147
Total Omission	2.6	0.0	0.0	0.0
Average Omission	9.9	13.1	19.1	15.5
Total Commission	32.7	19.18	12.74	17
Average Commission	58.4	66.11	49.26	56.35
AUC†	0.7188	0.6964	0.7401	0.7386
SE	0.0466	0.0817	0.2410	0.04
Z	90.94	4.4449	16.3284	16.6241

*N was divided into 50%training/50% testing for each model iteration

†AUC = area under the curve

### Predicted Distributions of *B. anthracis*

**Figure 1:**
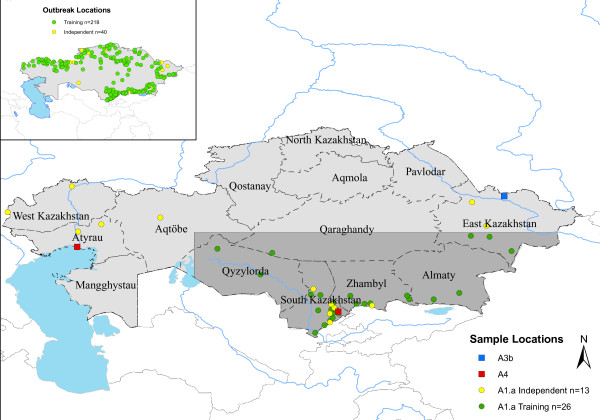
**Map of Kazakhstan with anthrax locality data**. (A) Locations of genotyped isolates. Inset shows locations of outbreaks used for the full outbreak model. Green points indicate training data for input into GARP and yellow points indicate independent points used to evaluate model accuracy. Shaded area indicates southern polygon used for creating projected models.

**Figure 2:**
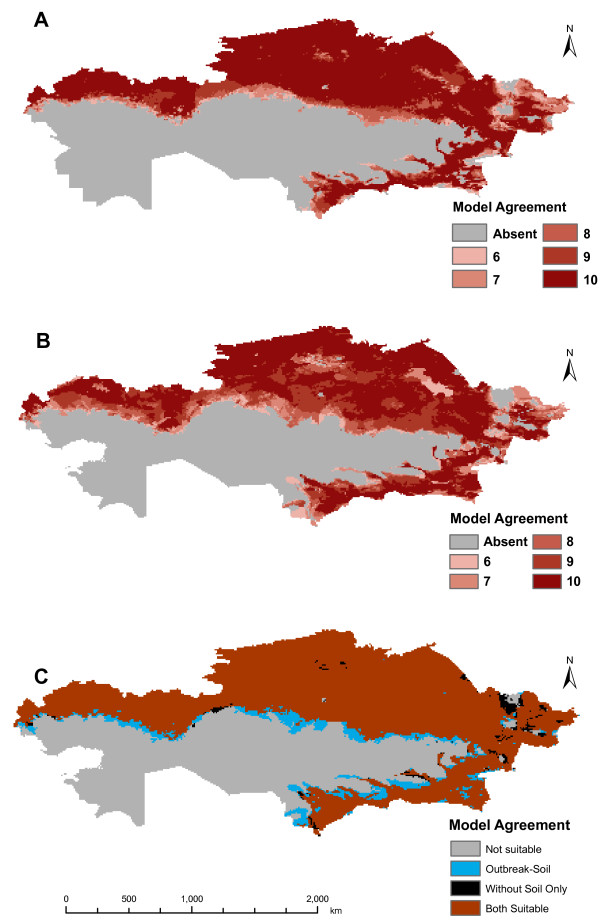
**Predicted distribution of *Bacillus anthracis *in Kazakhstan**. Predicted distribution of *Bacillus anthracis *in Kazakhstan based on outbreak data with and without soil variables. (A) Outbreak experiment (excluding soil variables) [26], (B) Outbreak-soil experiment (including soil variables), (C) Differences between distributions predicted by the two experiments.

**Figure 3:**
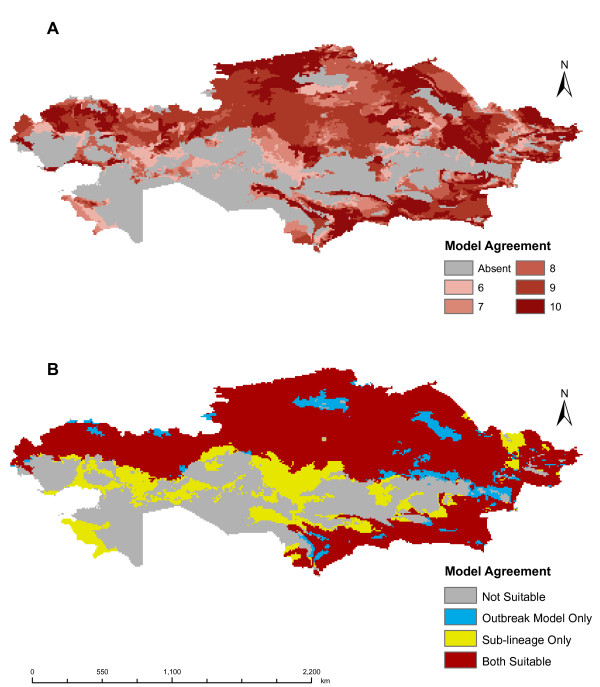
**Predicted geographic distribution of the *Bacillus anthracis *A1.a sub-lineage**. Comparison of predicted geographic distributions of *B. anthracis*. (A) distribution of *B. anthracis *predicted by the sub-lineage experiment, (B) difference between predicted distributions of the sub-lineage and the outbreak-soil experiments.

**Figure 4:**
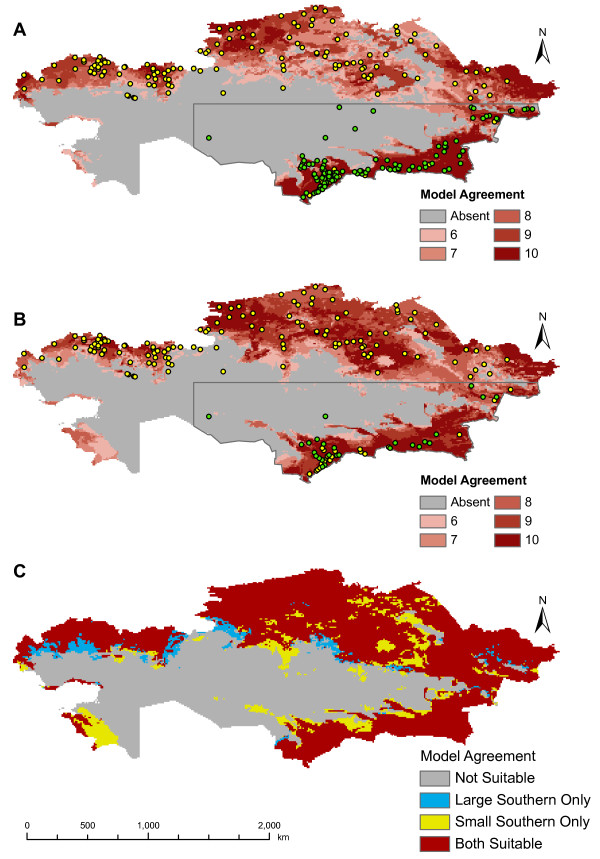
**Predicted geographic distribution of *B. anthracis *based on the large southern outbreak experiment and small southern outbreak experiment**. Predicted geographic distribution of *B. anthracis *based on (A) large southern outbreak experiment and (B) small southern outbreak experiment. (C) Difference between predicted geographic distributions. Green points indicate training data for input into GARP and yellow points indicate independent points used to evaluate model accuracy.

Locations used for input into GARP are shown in Figure 1. Based on areas of agreement of a minimum of six of the best subset models, the outbreak-soil experiment predicted *B. anthracis *across much of northern Kazakhstan and in a narrow band of the southeast. The interior of the country, which is primarily arid, was not predicted to be suitable for the pathogen. The results are similar to those of the experiment without the soil variables with respect to the geographic extent of areas of six or more best subset model agreement (Figure 2). The outbreak-soil experiment expanded two areas in the north which had lower model agreement. The A1.a sub-lineage experiment predicts a more extensive geographic distribution than that of the outbreak experiment, including areas in the northern interior and western portions of the country (Figure 3). The northern pockets of less suitable geographic areas seen in the outbreak-soil experiment were predicted to be unsuitable based on agreement of six or more best subset models. The overall extents of the geographic predictions of the two experiments were more similar in the south than in the north. The large and small southern outbreak experiments both predicted similar geographic extents as the outbreak-soil experiment (Figure 4). All three projected experiments (A1.a sub-lineage, large southern outbreak and small southern outbreak) were run ten additional times using random external data splits. The subsets of A1.a sub-lineage and small southern outbreak experiments showed greater degrees of spatial heterogeneity than did the large southern outbreak experiment set. (see Additional File 1: Random Subsets for illustration, available as a PDF file, and Additional File 2: Accuracy metrics for random subsets, available as a PDF file).

**Figure 5:**
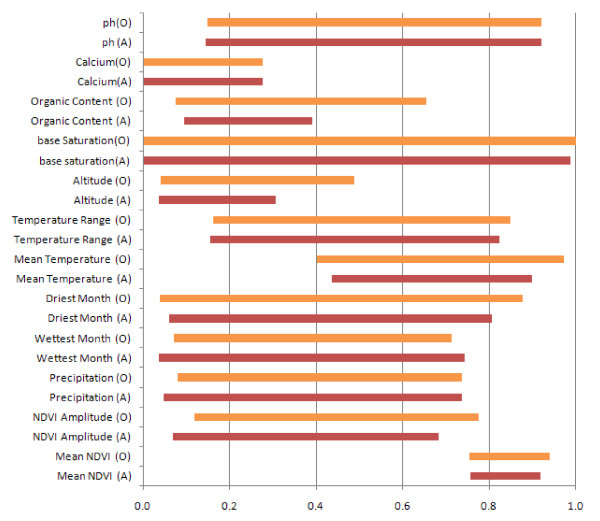
**Median ranges of environmental variables predicting *B. anthracis *presence by the outbreak-soil experiment**. O = Outbreak-Soil Experiment; A = A1.a sub-lineage experiment.

Each GARP model is composed of 50 if-then type rules (logic, range, negated range and atomic) which predict the presence or absence of the species for each pixel. Rule types for the ten best subset models from the outbreak-soil and A1.a sub-lineage experiments were extracted and are summarized in Table 3. Just over half of the outbreak-soil experiment rules were logit and no atomic rules were included, whereas range rules made up over 60% of the A1.a sub-lineage experiment rule types and this experiment included four atomic rules in the best subsets. Between 6 and 13 rules defined greater than 90% of areas predicted to be suitable for *B. anthracis *for each of the best subsets. Of the 95 rules which predicted the majority of the landscape in the outbreak-soil experiment, the majority (83%) were presence rules and of these 62% were range rules. The A1.a sub-lineage experiment had 99 total rules predict the majority of the landscape; all but one of these was a presence rule and 73% of the presence rules were range rules. The environmental tolerances described by the dominant rules suggest that mean NDVI, altitude, mean temperature, minimum soil calcium and minimum soil organic content are limiting variables for *B. anthracis *in Kazakhstan (Figure 5). Median minimum values of mean NDVI, NDVI amplitude, annual precipitation, dry month precipitation, wet month precipitation, mean temperature, altitude and soil organic content are significantly different between the A1.a sub-lineage and the outbreak-soil experiment using the Wilcox-Mann-Whitney test at a 95% significance level. Median maximum values of NDVI amplitude, mean temperature, dry month precipitation, altitude, soil base saturation and soil organic content differ between the two experiments.

**Table 3:** Rules types from ten best models of the outbreak-soil and A1.a sub-lineage experiments. Values shown are number of rule types in the rule set (column %)

	Outbreak-Soil Rule Set										
**Rule Type**	**2**	**6**	**13**	**21**	**24**	**25**	**29**	**39**	**48**	**51**	**Total**

Logit	17(34)	32(64)	34(68)	25(50)	22(44)	26(52)	36(72)	35(70)	25(50)	27(54)	279(55.8)
Negated Range	2(4)	1(2)	0(0)	0(0)	2(4)	2(4)	0(0)	1(2)	7(14)	7(14)	22(4.4)
Range	31(62)	17(34)	16(32)	25(50)	26(52)	22(44)	14(28)	14(28)	18(36)	16(32)	199(39.8)

	**A1.a Sub-lineage Rule Set**										

**Rule Type**	**1**	**10**	**21**	**40**	**49**	**51**	**54**	**76**	**91**	**93**	**Total**

Atomic	1(2)	2(4)	1(2)	0(0)	0(0)	0(0)	0(0)	0(0)	0(0)	0(0)	4(0.8)
Logit	15(30)	5(10)	22(44)	20(40)	30(60)	2(4)	7(14)	24(44)	22(44)	9(18)	156(31.2)
Negated Range	10(20)	0(0)	4(8)	2(4)	0(0)	0(0)	0(0)	7(14)	0(0)	0(0)	23(5.6)
Range	24(48)	43(86)	23(46)	28(56)	20(40)	48(96)	43(86)	19(38)	28(56)	41(82)	317(63.4)

**Figure 6:**
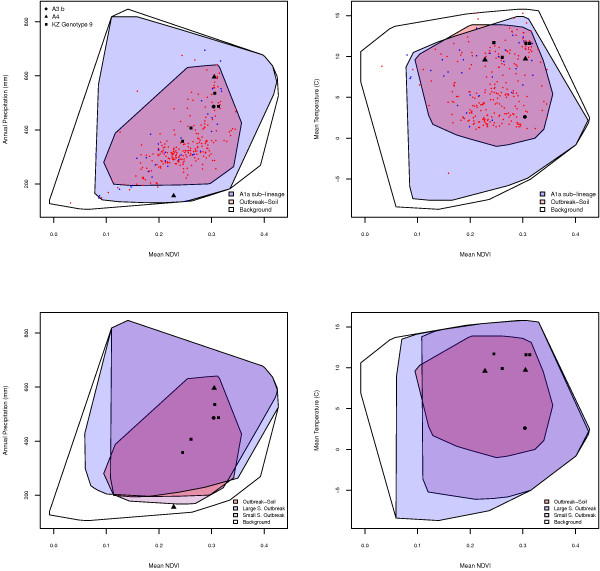
**Distribution of *B. anthracis *in ecological space**. Predicted distribution of *B. anthracis *in ecological space based on areas of ten best subset model agreement. Red points = outbreak locations, blue points = A1.a isolate locations, black triangles = A4 locations, black circle = A3.b location.

Values of the limiting variables were extracted from areas of ten best subset model agreement and plotted in two dimensional variable space. The A1.a sub-lineage experiment showed a broader ecological envelope than the outbreak-soil experiment based on areas of ten best subset model agreement, despite the smaller geographic area predicted by agreement of all ten models (Figure 6). The two A4 locations, which are distant from each other geographically, are found within a narrow range of mean NDVI and mean temperature, but occupy nearly opposite ends of the range of precipitation values. Finally, the A3.b location was associated with ecological conditions towards the outer boundaries of the ecological envelope predicted by the outbreak-soil experiment.

## Discussion

This study assesses the addition of soil variables to a previously developed ecological niche model for *Bacillus anthracis *and is the first known to model the ecological and geographic distribution of a single sub-lineage of *B. anthracis*. Inclusion of available soil variables into our anthrax outbreak model resulted in subtle changes in the likelihood of the pathogen in areas of northern Kazakhstan, but did not substantially change the extent of geographic predictions or results of rule set analyses [26]. The areas predicted as less suitable by the outbreak-soil model correspond to regions of locally different values for all four soil variables (see Additional File 3: Soil Variables, available as a PDF document). However, it is not known whether these areas represent a unique ecological region or if measurement in these areas was affected by error or bias. The low minimum soil calcium association found in the rule set analyses contrasts with previous literature suggesting that *B. anthracis *spore persistence is associated with high soil calcium levels [3,30]. The results of our rule set analysis, however, are not directly comparable to previous work in that different units of measurement and sampling techniques were used. In addition, the soils data available for this study had a relatively coarse resolution of 1 km and were further aggregated to 8 km to match other climatic data for model development. As a result, fine resolution relationships between soil and anthrax occurrences would likely be missed in this experiment. Improved resolution of soil and outbreak data, such as exact carcass locations, are likely necessary to characterize the role of soil parameters in promoting anthrax spore persistence [3,31] and for better understanding the spatio-temporal dynamics and ecology of local outbreaks [32].

The A1.a sub-lineage experiment predicted a more extensive geographic area of anthrax presence than did the outbreak experiment. This is most pronounced in the northern and central portions of the country. The median minimum values of most variables defined by the dominant rule sets were significantly different. When ecological values of limiting variables were extracted from geographic areas of best subset agreement and plotted in two dimensional variable-space, the A1.a sub-lineage was associated with a larger ecological envelope than the outbreak-soil data. This finding illustrates that analysis of dominant rule sets alone should be interpreted with some caution. The variable ranges derived from the dominant rule sets summarize approximately only one-fifth (or fewer) of the total number of rules generated by GARP in the ten best subsets. The values extracted from predicted areas on the landscape are derived from all 500 rules contained in the 10 best subsests and represent the spectrum of complex interactions between variables and the landscape. Because geographic areas of model agreement can be thought of as representative of all sub-sampled regions or populations [33], the finding of a larger ecological envelope for the A1.a sub-lineage experiment lends support to the hypothesis that the A1.a sub-lineage of *B. anthracis *may have broad environmental tolerances that influence its broad geographic distribution [3,24].

The A lineage is more widely distributed globally than other subtypes, perhaps reflecting a greater level of fitness as compared to other lineages [24]. This finding has been shown on a local scale as well. Isolates of the A lineage in Kruger National Park, South Africa, as defined by MLVA-8 typing, were more diffusely distributed and showed a distinctly different spatial cluster pattern than those of the B lineage. Furthermore, the B lineage isolates occupied a narrow range of available ecological conditions within those occupied by the A lineage isolates [3]. In the experiments reported here, the A1.a sub-lineage locations were associated with lower pH values than the outbreak locations and this could provisionally support the findings from Kruger National Park [3]. It follows that that a *B. anthracis *lineage and/or sub-lineage other than A1.a may predominate in the northern regions of Kazakhstan and is driving the narrower geographic and ecological prediction of the outbreak-soil experiment. Genotyping of additional isolates from northern Kazakhstan is necessary to evaluate this hypothesis. Locations of outbreaks of the A4 lineage in geographical and ecological space suggests that this genotype may also have a relatively broad distribution, which is consistent with the A4 sub-lineage being found across the Middle East and China [29,34]. Although we cannot make inferences regarding the environmental affinities of the single A3.b isolate, we note it is located on the far eastern border of Kazakhstan in an area predicted to be unsuitable for anthrax by the outbreak-soil experiment. The A3.b sub-lineage has been isolated from geographically limited areas, most notably northern China and Texas, and the presence of this genotype is likely a result of historical trade routes [24,34,35].

The genetic diversity of *B. anthracis *isolates in southern Kazakhstan is not surprising given the location of this area along the historic Silk Road [29], but this diversity also implies that this region is supportive of spore persistence. Associations between genotypes of *B. anthracis*, environment, virulence or host species have not yet been fully explored and it is unknown whether genotype influences epidemiological characteristics of outbreaks. Understanding these relationships will improve our understanding of anthrax disease ecology, help focus surveillance and efficiently direct proactive vaccination. Furthermore, this knowledge can help distinguish between naturally occurring outbreaks, contamination and potential bioterrorism, and greatly enhance epidemiological trace back (tracing outbreaks to source) efforts during outbreaks.

Few studies using GARP have quantified and examined rule types. GARP begins creating rule sets by choosing the first rule type at random and successful rules are carried forward into subsequent rule sets. Thus, the first rule type chosen at random will often predominate in the final rule sets. Blackburn and Joyner et. al. both presented summaries and distribution of the dominant rule types predicting *B. anthracis *in the continental United States and Kazakhstan, respectively [26,32]. Blackburn noted a predominance of range rules among a total of 63 rules predicting greater than 90% of the landscape [32]. Joyner et al divided Kazakhstan into northern and southern halves and modelled the two sections separately [11]. A greater percentage of range rules described the northern half of the country, whereas logit rules predominantly described the southern half of the country. Here, logit rules dominated in the out-break soil experiment and range rules in the A1.a sub-lineage experiment. Further work is required to tease out whether dominant rule types result from the stochastic nature of GARP or are related to complex interactions between the organism and environmental variables. Additional future work should also explore how rule sets and ecological values found over predicted areas of the landscape can be used to enhance our understanding of the ecology of an organism.

Several authors have noted that ENM-based predictions of species with widespread distributions show reduced model accuracy which can be improved by dividing species or the range into sub-units [33,36-39]. One explanation for the apparent poor accuracy of models of widespread species is the use of AUC. The AUC is sensitive to the area predicted to be suitable for a species relative to the total land area analyzed [33,40]. Other considerations include non-uniformity of presence locations (geographical bias), and biological factors such as local ecological adaptations and genetic diversity [33,37-39]. For example, modelling of *Francisella tularensis *genotypes in the US yielded overlapping, yet different, geographic predictions and ecological associations [12]. Interestingly, this difference was apparent at intermediate, as opposed to coarse, phylogenetic levels. Similarly, Fisher et. al. showed that three genotype categories of the broadly distributed pathogenic fungus *Penicillium marneffei *correlated with environmental heterogeneity across Vietnam [21]. Using GARP, the genotypes classes were predicted to occupy three non-overlapping geographic areas. As a consequence of *B. anthracis *being a widely distributed species, models of anthrax outbreaks are subject to similar limitations in accuracy. Genotype specific models may therefore have improved accuracy and predictive power, and should be explored at multiple phylogenetic levels.

It is worthwhile to evaluate modelling limitations which could potentially explain differences between experiments. Despite studies showing GARP to be robust when predicting new landscapes [14,25,41], our use of a projected modelling strategy may wrongly predict geographic and ecological distribution given the large geographic area in question. However, projected models created using southern outbreak points show similar geographic predictions as the outbreak-soil experiment, supporting that the broader geographic A1.a sub-lineage prediction is not simply an artefact of the modelling technique. That the large and small southern outbreak experiments showed a lesser degree of model agreement than the outbreak-soil experiment and that the large outbreak subsetting procedure had improved spatial homogeneity over the small southern outbreaks subsetting indicates that projected models may be sensitive to issues of sample size and clustering. Issues relating to sample size appear to be important in our study. The predicted geographic distribution of *B. anthracis *varied among the 10 random data splits for both the Al.a sub-lineage and small southern outbreak experiments. In contrast, the same random data splitting procedure performed with the large southern outbreak and the outbreak-soil experiments showed spatial homogeneity among models [26]. Although Stockwell and Peterson and Hernandez found that as few as 10 presence points are adequate for accurate GARP models [36,39], additional work has shown that certain species or geographic scenarios are more sensitive to sample size than others and that model accuracy can be sensitive to both sample size and extent of the species' range [42]. Here, any effect of sample size is likely exaggerated by projection onto a large geographic area and the relatively limited resolution the data [36,42].

Some additional limitations apply to our findings. The A1.a sub-lineage isolate collection was derived from multiple species, including livestock and humans, and from soil samples. The outbreak data, however, were derived from livestock only. The impact of this on model comparisons is unknown since associations between host, genotype and environment are as yet unexplored. We are limiting our modelled area by political boundaries as opposed to biogeographic limits. Finally, the genotyped isolate collection is geographically biased towards the southeast portion of Kazakhstan and spans a relatively long period of time [29]. Sampling bias is common in niche models using historical collections and can create artificial patterns in the data, although GARP is arguably less sensitive than other modelling algorithms to spatial bias [10,37,43,44]. Future genotyping of additional isolates from under-sampled portions of the country, particularly the northern oblasts, will be essential to better characterize the genetic diversity and ecology of anthrax in Kazakhstan, allowing the construction of more refined predictive models.

## Conclusions

The inclusion of available soil variables resulted in subtle changes in the predicted geographic distribution of anthrax in Kazakhstan, but the experiment is limited by the nature of available soil variables. Standardized soil variables and finer resolution data will be essential to characterizing the importance of soil parameters in *B. anthracis *persistence. The A1.a sub-lineage experiment showed a larger geographic and ecological distribution than the outbreak based experiment. Understanding genetic-environmental associations will be essential to accurate modelling of anthrax for use in disease prevention and control in Kazakhstan

## Methods

### Anthrax occurrence data

Presence points for outbreak-based models were taken from the database created by Joyner et.al. [26]. Briefly, historical records were used to construct a database of 3,947 anthrax outbreaks reported in Kazakhstan between 1937 and 2006. The data were sequentially filtered to create a dataset containing the latitude and longitude of outbreaks in cattle, sheep and goats which occurred between 1960 and 2000. This time period reflects the implementation of mass vaccination and corresponds to the averaged data from both the WorldClim and soils data sets [45,46]. The final dataset contained 258 spatially unique points, meaning that only one outbreak point occurred in each 8 km^2 ^pixel. An 8 km^2 ^resolution was chosen because outbreaks were mapped to the nearest village and some outbreaks occurred greater than 1 km from the village coordinates. This data set is hereafter referred to as the full outbreak dataset.

A second dataset was constructed using outbreak isolates genotyped by Aikimbayev et.al. [29]. Isolates were grouped into the A1.a (n = 78), A3.b (n = 6) and A4 (n = 4) sub-lineages using unweighted pair group method with arithmetic mean (UPGMA) cluster analysis and the 89 *B. anthracis *genotypes identified by Keim et.al. [22]. This was filtered to contain only spatially unique points at a resolution of 8 km^2 ^resulting in 42 spatially unique points, of which 39 were A1.a, two were A4 and 1 was A3 b. Locality data are mapped in Figure 1. Only the A1.a sub-lineage had an adequate number of spatially unique points for modelling. The A1.a locations were geographically biased towards the south-eastern portion of the country. To reduce the problem of largely unsampled areas being considered as absence points, we created a polygon encompassing southeast Kazakhstan using latitude 48 N and 60 E as the northern and western boundaries, respectively, and the country's political boundaries in the south and east. The boundaries of this southern polygon were derived from examination of the locations of the A1.a isolates and from the different northern and southern ecological associations noted by Joyner [26]. The southern polygon was used to clip the A1.a locality points in ArcMap and this set of southern A1.a locations was used for the sub-lineage experiment. The same procedure was used to create a southern outbreak dataset.

### Environmental Data

We used six environmental coverages downloaded from the WorldClim website http://www.worldclim.org[46]. The WorldClim variables are calculated from interpolation of monthly temperature and precipitation measurements recorded at stations located worldwide between 1961 and 2000. Monthly values are transformed by WorldClim into 19 bioclimatic variable grids that describe annual trends, seasonality and potentially limiting ecological parameters such as temperature of the coldest and warmest months. Two satellite-derived environmental variables describing temperature and vegetation measures were obtained from the Trypanosomiasis and Land Use in Africa (TALA) research group (Oxford, United Kingdom) [47].

We added four soil variables to the set of eight environmental layers used in the previous study to test the influence of soil parameters on the outbreak model. Soil variables were derived from the Harmonized World Soil Database and were available at 1 km^2 ^resolution [45]. All coverages were re-sampled to 8 km^2 ^and clipped to the boundaries of Kazakhstan in ArcView 3.3. (Environmental Systems Research institute, Redlands, CA). An identical set of coverages was clipped to the southern polygon. The final set of coverages is given in Table 4.

**Table 4:** Environmental coverages used for GARP models

Environmental Variable (unit)	Name	Source
Elevation (m)	Altitude	WorldClim*
Annual Temperature Range (°C)	BIO7	WorldClim
Annual Mean Temperature (°C)	BIO1	WorldClim
Precipitation of Driest Month (mm)	BIO14	WorldClim
Precipitation of Wettest Month (mm)	BIO13	WorldClim
Annual Precipitation (mm)	BIO12	WorldClim
NDVI Amplitude (no units)	wd1014 a1	TALA†
Mean NDVI (no units)	wd1014 a0	TALA
Soil pH (-log(H⁺))		HWSD‡
Topsoil Calcium (% weight)		HWSD
Topsoil Organic Content (% weight)		HWSD
Subsoil Base Saturation (%)		HWSD

*http://www.worldclim.org[46]

† Trypanosomiasis and Land Use in Africa (TALA) research group [47]

‡Harmonized World Soil Database [45]

### Ecological Niche Modelling

This study used the Genetic Algorithm for Rule-Set Prediction (GARP) to perform the ecological niche modelling [48]. Models were developed in Desktop GARP v.1.1.3, which gives the user the option to write out the rule sets for each model. Briefly, GARP is a presence only modelling technique that detects non-random associations between species localities and specific environmental variables. Through an iterative process, relationships are expressed as a series of logic statements, or rules, of which there are four types: (1) logit - based on logistic regression; (2) atomic - single value for a given variable that predicts presence; (3) range - a range of values of a given variable that predicts presence; and (4) negated range - a range of values outside of which presence is predicted. Each individual GARP model is a set of 50 rules that are randomly generated, tested and modified. The user sets a maximum number of models to be created in a single experiment. A best subsets procedure within GARP then selects a set of optimal models based on user defined omission and commission criteria [48]. The algorithm is a two-step process, where first relationships are defined in variable space through a random walk and then applied to the geographic landscape where those conditions are met [25]. GARP therefore has the benefit of being able to project rule sets onto the environmental layers of a different landscape and has been shown to be robust in this application [25,41].

### Model building and evaluation

To test the effect of soils on the outbreak experiment we used the full outbreak dataset as locality points and the twelve environmental variables described in Table 3. The 258 spatially unique points were randomly divided into an 85% (n = 218) training set used for model building and a 15% (n = 39) testing set for model evaluation. The 32 southern A1.a points were divided into an 80% (n = 26) training set and a 20% (n = 6) testing set in order to maximize points available for testing [33,39,42]. The A1.a training set was input into GARP with the set of environmental coverages clipped to the southern polygon for model development. Rules from this southern A1.a experiment were projected onto the entire landscape of Kazakhstan. In order to test the robustness of the A1.a model projections given the relatively small sample size and issues of transferability, two experiments using the southern outbreak data were performed. The first utilized all 142 southern outbreak points and is referred to as the large southern outbreak experiment. For the second, 32 points were randomly selected from the southern outbreak dataset (small southern outbreak experiment). For these experiments an 85%/15% and 80%/20%, respectively, external data split was performed and the experiments conducted as for the sub-lineage. The four experiments are summarized in Table 4.

For all niche modelling experiments, we specified 200 models with a maximum of 1,000 iterations and a convergence limit of 0.01. The training data were input into GARP with a 50% training/50% testing internal data partition. The best subset procedure selected the best 20 models under a 10% hard omission threshold and a 50% commission threshold. The resulting ten best subset models were imported in ArcGIS and summated using the raster calculator function of the Spatial Analyst extension. This created a single cumulative raster file of model agreement for *B. anthracis *presence ranging from 0 (all models predict absence) to 10 (all models predict presence). The more models that predict presence for a given pixel, the higher the likelihood that the pixel can support *B. anthracis*.

Rule types from the ten best models of the outbreak-soil experiment and the A1.a sub-lineage experiment were extracted with a python script (K.M. McNyset, US NOAA) and summarized to illustrate the relative numbers of each rule type. Dominant rules, or the subset of rules that together predict over 90% of the landscape, for each model were identified. We extracted the minimum and maximum values of range rules using the python script. When logit rules were identified, we extracted the range of values across the pixels predicted by that rule using the "Extract Values to Points" routine of the Spatial Analyst extension in ArcMap. Median minimum and maximum values for each variable were calculated in SAS (SAS 9.2, Cary, N.C.) and plotted as a bar graph. Differences in median and maximum values between experiments were assessed using Wilcoxon-Mann_Whitney test in SAS.

Predictive performance of the best subset models was evaluated with an area under the curve (AUC) in a receiver operating characteristic (ROC) analysis using the independent test data withheld from the original datasets [40]. For the projected models, testing points included presence points outside the southern polygon in addition to points withheld from within the southern polygon. Values of AUC, which range from 0.5 (no different from random) to 1 (a perfect model), are derived from measures of sensitivity (absence of omission error) and specificity (absence of commission error). The calculated value is compared to that of a random model using a z-test. In addition, measures of omission and commission were calculated using the summed ten best subset models. Total and average omission values evaluate how well GARP predicts the presence of known locality points not included in the model building data. Total and average commission is the percent of pixels predicted as presence by the summated model and the average of this value for all ten best subset models, respectively. Large variation between the two measures of commission suggests substantial variation between the proportions of the landscape predicted present by each of the ten best subset models [33].

Environmental values of 5,000 randomly chosen points from areas predicted by all 10 of the best subset models were extracted using the "Extract Values to Points" routine of the Spatial Analyst extension in ArcMap. Values of each environmental variable at each presence point and at 5,000 random points representing the total available environmental space (background) were similarly extracted. Specific environmental values appearing to be limiting factors for prediction of *B. anthracis *were chosen based on the rule set evaluation (Figure 5) and visualized in 2-dimensional ecological space against the background of available environmental conditions using R 2.1.1 http://www.R-project.org.

## Authors' contributions

JCM planned the study, ran the experiments and wrote the manuscript. JKB contributed to planning and running the experiments and drafting the manuscript. LL, YP, JKB constructed the GIS database for outbreaks and genotyped strains. LL, YP, AA collected and managed the *Bacillus anthracis *strains. MVE, LL, YP, AA genotyped the strains. All authors reviewed the final draft of the manuscript.

## Supplementary Material

Additional file 1 **Random Subsets**. Predicted geographic distribution of B. anthracis based on 10 random subsets of input locality points for the Aa.1 sub-lineage, large southern outbreak and small southern outbreak experiments. Click here for file

Additional file 2 **Accuracy metrics for random subsets**. Accuracy metrics of 10 random subsets of input locality points for the Aa.1 sub-lineage, large southern outbreak and small southern outbreak experiments Click here for file

Additional file 3 **Soil Variables**. Mapped values of the four soil variables (minimum soil pH, minimum soil organic content, minimum soil calcium and minimum soil base saturation). Click here for file

## References

[B1] Hugh-Jones M Blackburn J The ecology of Bacillus anthracis Molecular Aspects of Medicine 2009 30 356 367 10.1016/j.mam.2009.08.003 19720074

[B2] Dragon DC Rennie RP The ecology of anthrax spores: tough but not invincible The Canadian Veterinary Journal 1995 36 295 PMC1686874 7773917

[B3] Smith KL DeVos V Bryden H Price LB Hugh-Jones ME Keim P Bacillus anthracis diversity in Kruger National Park J Clin Microbiol 2000 38 3780 3784 11015402 10.1128/jcm.38.10.3780-3784.2000 PMC87475

[B4] Van Ness G Stein CD Soils of the United States favorable for Anthrax J Am Vet Med Assoc 1956 128 7 9 13278269

[B5] Van Ness G Ecology of Anthrax Science 1971 172 1303 1307 10.1126/science.172.3990.1303 4996306

[B6] Epp T Waldner C Argue CK Case-control study investigating an anthrax outbreak in Saskatchewan, Canada--Summer 2006 Can Vet J 2010 51 973 978 21119863 PMC2920171

[B7] Parkinson R Rajic A Jenson C Investigation of an anthrax outbreak in Alberta in 1999 using a geographic information system Can Vet J 2003 44 315 318 12715984 PMC372251

[B8] Bales ME Dannenberg AL Brachman PS Kaufmann AF Klatsky PC Ashford DA Epidemiologic Responses to Anthrax Outbreaks: A Review of Field Investigations, 1950-2001 Emerging Infectious Diseases 2002 8 1163 12396934 10.3201/eid0810.020223 PMC2730298

[B9] Woods CW Ospanov K Myrzabekov A Favorov M Plikaytis B Ashford DA Risk factors for human anthrax among contacts of anthrax-infected livestock in Kazakhstan The American Journal of Tropical Medicine and Hygiene 2004 71 48 15238688

[B10] Blackburn JK McNyset KM Curtis A Hugh-Jones ME Modeling the geographic distribution of Bacillus anthracis, the causative agent of anthrax disease, for the contiguous United States using predictive ecologic niche modeling American Journal of Tropical Medicine and Hygiene 2007 77 1103 1110 18165531

[B11] Joyner TA Lukhnova L Pazilov Y Temiralyeva G Hugh-Jones ME Aikimbayev A Blackburn JK Modeling the potential distribution of Bacillus anthracis under multiple climate change scenarios for Kazakhstan PLoS One 2010 5 e9596 10.1371/journal.pone.0009596 20231894 PMC2834750

[B12] Nakazawa Y Williams RA Peterson AT Mead PS Kugeler KJ Petersen JM Ecological niche modeling of Francisella tularensis subspecies and clades in the United States Am J Trop Med Hyg 2010 82 912 918 10.4269/ajtmh.2010.09-0354 20439975 PMC2861374

[B13] Peterson AT Bauer JT Mills JN Ecologic and geographic distribution of filovirus disease Emerging Infectious Diseases 2004 10 40 47 15078595 10.3201/eid1001.030125 PMC3322747

[B14] Ron SR Predicting the Distribution of the Amphibian Pathogen Batrachochytrium dendrobatidis in the New World1 Biotropica 2005 37 209 221 10.1111/j.1744-7429.2005.00028.x

[B15] Adjemian JCZ Girvetz EH Beckett L Foley JE Analysis of Genetic Algorithm for Rule-Set Production (GARP) modeling approach for predicting distributions of fleas implicated as vectors of plague, Yersinia pestis, in California J Med Entomol 2006 43 93 103 10.1603/0022-2585(2006)043[0093:AOGAFR]2.0.CO;2 16506453

[B16] Costa J Peterson AT Beard CB Ecologic niche modeling and differentiation of populations of Triatoma brasiliensis neiva, 1911, the most important Chagas' disease vector in northeastern Brazil (hemiptera, reduviidae, triatominae) American Journal of Tropical Medicine and Hygiene 2002 67 516 520 12479554 10.4269/ajtmh.2002.67.516

[B17] Peterson AT Shaw J Lutzomyia vectors for cutaneous leishmaniasis in Southern Brazil: ecological niche models, predicted geographic distributions, and climate change effects Int J Parasitol 2003 33 919 931 10.1016/S0020-7519(03)00094-8 12906876

[B18] Peterson AT Sanchez-Cordero V Ben Beard C Ramsey JM Ecologic niche modeling and potential reservoirs for Chagas disease, Mexico Emerging Infectious Diseases 2002 8 662 667 12095431 10.3201/eid0807.010454 PMC2730326

[B19] Grinnell J The niche-relationships of the California Thrasher Auk 1917 34 427 433

[B20] Hutchinson GE Population Studies - Animal Ecology and Demography - Concluding Remarks Cold Spring Harb Sym 1957 22 415 427

[B21] Fisher MC Hanage WP De Hoog S Johnson E Smith MD White NJ Vanittanakom N Low effective dispersal of asexual genotypes in heterogeneous landscapes by the endemic pathogen Penicillium marneffei PLoS Pathogens 2005 1 1986 1990 10.1371/journal.ppat.0010020 PMC1266309 16254598

[B22] Keim P Price L Klevytska A Smith K Schupp J Okinaka R Jackson P Hugh-Jones M Multiple-locus variable-number tandem repeat analysis reveals genetic relationships within Bacillus anthracis Journal of Bacteriology 2000 182 2928 10.1128/JB.182.10.2928-2936.2000 10781564 PMC102004

[B23] Lista F Faggioni G Valjevac S Ciammaruconi A Vaissaire J Le Doujet C Gorgé O De Santis R Carattoli A Ciervo A Genotyping of Bacillus anthracis strains based on automated capillary 25-loci multiple locus variable-number tandem repeats analysis BMC Microbiology 2006 6 33 10.1186/1471-2180-6-33 16600037 PMC1479350

[B24] Van Ert MN Easterday WR Huynh LY Okinaka RT Hugh-Jones ME Ravel J Zanecki SR Pearson T Simonson TS U'Ren JM Global Genetic Population Structure of Bacillus anthracis PLoS One 2007 2 10.1371/journal.pone.0000461 PMC1866244 17520020

[B25] Blackburn J O'Connel K, et al Integrating Geographic Information Systems and Ecological Niche Modeling into Disease Ecology: A Case Study of Bacillus anthracis in the United States and Mexico Emerging and Endemic Pathogens 2010 Springer Science 59 88

[B26] Joyner Ecological niche modeling of a zoonosis: a case study using anthrax outbreaks and climate change in Kazakhstan 2010 University of Florida

[B27] Cherkasskiy BL A national register of historic and contemporary anthrax foci J Appl Microbiol 1999 87 192 195 10.1046/j.1365-2672.1999.00868.x 10475946

[B28] Hugh-Jones M 1996-97 Global Anthrax Report J Appl Microbiol 1999 87 189 191 10.1046/j.1365-2672.1999.00867.x 10475945

[B29] Aikembayev AM Lukhnova L Temiraliyeva G Meka-Mechenko T Pazylov Y Zakaryan S Denissov G Easterday WR Van Ert MN Keim P Historical distribution and molecular diversity of Bacillus anthracis, Kazakhstan Emerg Infect Dis 2010 16 789 796 20409368 10.3201/eid1605.091427 PMC2953997

[B30] Himsworth CG The danger of lime use in agricultural anthrax disinfection procedures: the potential role of calcium in the preservation of anthrax spores Can Vet J 2008 49 1208 1210 19252713 PMC2583417

[B31] Van Ness G Geologic Implications of Anthrax The Geological Society of America Special Paper 1967 90 61 64

[B32] Blackburn JK Evaluating the spatial ecology of anthrax in North America: Examining epidemiological components across multiple geographic scales using a GIS-based approach 2006 Louisiana State University

[B33] McNyset KM Use of ecological niche modelling to predict distributions of freshwater fish species in Kansas Ecol Freshw Fish 2005 14 243 255 10.1111/j.1600-0633.2005.00101.x

[B34] Simonson TS Okinaka RT Wang B Easterday WR Huynh L U'Ren JM Dukerich M Zanecki SR Kenefic LJ Beaudry J Bacillus anthracis in China and its relationship to worldwide lineages BMC Microbiology 2009 9 71 10.1186/1471-2180-9-71 19368722 PMC2674057

[B35] Kenefic LJ Pearson T Okinaka RT Chung WK Max T Trim CP Beaudry JA Schupp JM Van Ert MN Marston CK Texas isolates closely related to Bacillus anthracis Ames Emerg Infect Dis 2008 14 1494 1496 10.3201/eid1409.080076 18760033 PMC2603087

[B36] Hernandez PA Graham CH Master LL Albert DL The effect of sample size and species characteristics on performance of different species distribution modeling methods Ecography 2006 29 773 785 10.1111/j.0906-7590.2006.04700.x

[B37] Peterson AT Predicting species'geographic distributions based on ecological niche modeling The Condor 2001 103 599 605 10.1650/0010-5422(2001)103[0599:PSGDBO]2.0.CO;2

[B38] Rice NH Martinez-Meyer E Peterson AT Ecological niche differentiation in the Aphelocoma jays: a phylogenetic perspective Biol J Linn Soc 2003 80 369 383 10.1046/j.1095-8312.2003.00242.x

[B39] Stockwell DRB Peterson AT Effects of sample size on accuracy of species distribution models Ecological Modelling 2002 148 1 13 10.1016/S0304-3800(01)00388-X

[B40] Wiley E McNyset KM Peterson AT Robins CR Stewart AM Niche modeling and geographic range predictions in the marine environment using a machine-learning algorithm Oceanography 2003 16 120 127

[B41] Peterson AT Pape M Eaton M Transferability and model evaluation in ecological niche modeling: a comparison of GARP and Maxent Ecography 2007 30 550 560

[B42] Peterson AT Ball LG Cohoon KP Predicting distributions of Mexican birds using ecological niche modelling methods Ibis-Journal of the British Ornithologists Union 2002 144 27

[B43] Araujo MB Guisan A Five (or so) challenges for species distribution modelling J Biogeogr 2006 33 1677 1688 10.1111/j.1365-2699.2006.01584.x

[B44] Stockwell D Peters D The GARP modelling system: problems and solutions to automated spatial prediction Int J Geogr Inf Sci 1999 13 143 158 10.1080/136588199241391

[B45] FAO/IIASA/ISRIC/ISSCAS/JRC Harmonized World Soil Database (version 1.1) 2009 Rome, Italy and IIASA, Laxenburg, Austria

[B46] Hijmans RJ Cameron SE Parra JL Jones PG Jarvis A Very high resolution interpolated climate surfaces for global land areas Int J Climatol 2005 25 1965 1978 10.1002/joc.1276

[B47] Hay SI Tatem AJ Graham AJ Goetz SJ Rogers DJ Global environmental data for mapping infectious disease distribution Adv Parasit 2006 62 37 77 10.1016/S0065-308X(05)62002-7 PMC3154638 16647967

[B48] Anderson RP Lew D Peterson AT Evaluating predictive models of species' distributions: criteria for selecting optimal models Ecological Modelling 2003 162 211 232 10.1016/S0304-3800(02)00349-6

