# Supplementary material for: Ecological Niche Modelling of the Bacillus anthracis A1.a sub-lineage in Kazakhstan
Source: BMC Ecol. 2011 Dec 12;11:32. doi: 10.1186/1472-6785-11-32 (PMC3260114; doi:10.1186/1472-6785-11-32)

A1.a Sub-lineage Random 80%/20% Subsets

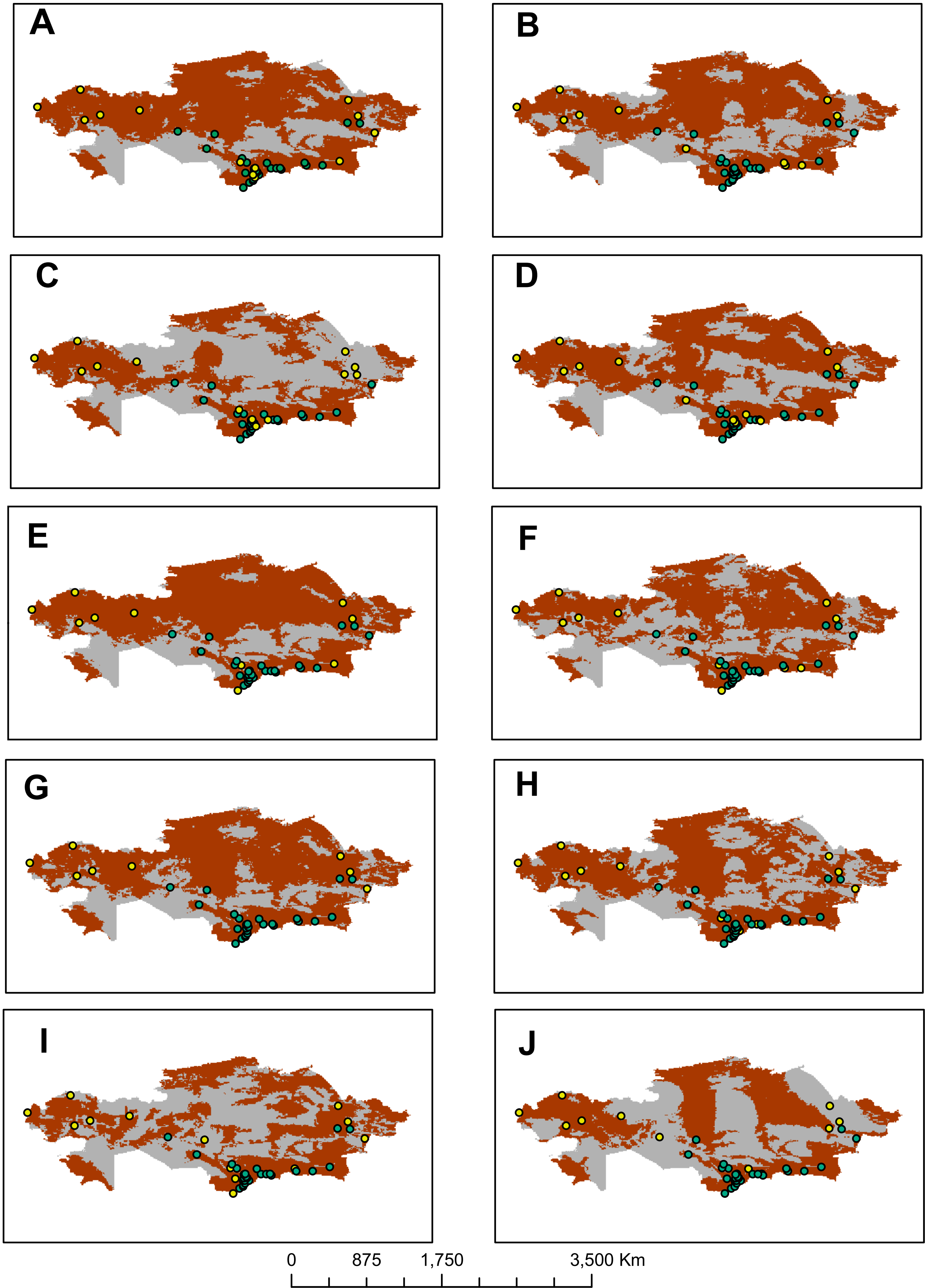

Small Southern Outbreak 80%/20% Random Subsets

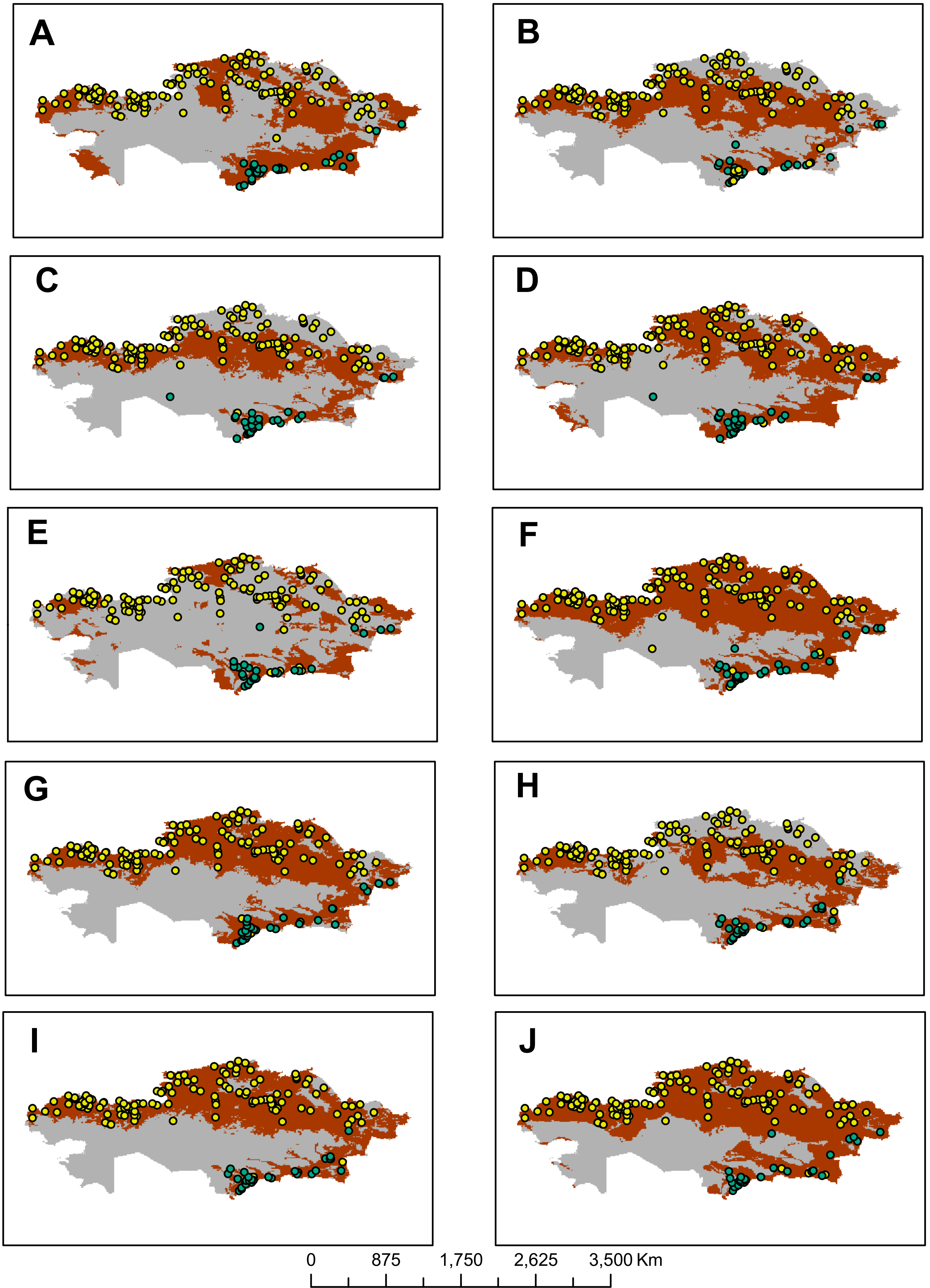

Large Southern Outbreak Random 85%/15% Subsets

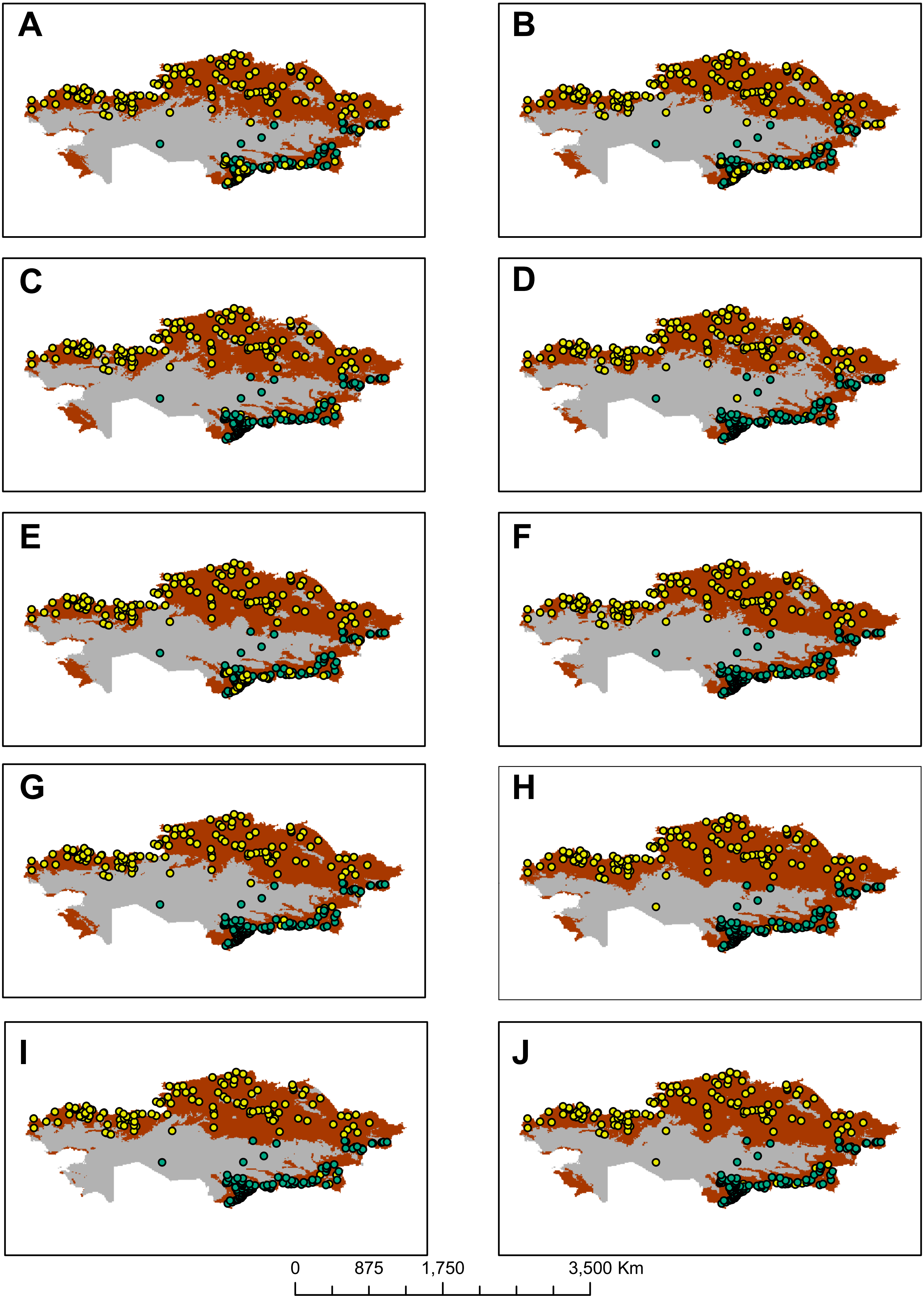

Supplement: Additional file 1 — Random Subsets. Predicted geographic distribution of B. anthracis based on 10 random subsets of input locality points for the Aa.1 sub-lineage, large southern outbreak and small southern outbreak experiments. [file 1472-6785-11-32-S1.PDF]
