# Supplementary material for: Ecological Niche Modelling of the Bacillus anthracis A1.a sub-lineage in Kazakhstan
Source: BMC Ecol. 2011 Dec 12;11:32. doi: 10.1186/1472-6785-11-32 (PMC3260114; doi:10.1186/1472-6785-11-32)
Supplement: Additional file 2 — Accuracy metrics for random subsets. Accuracy metrics of 10 random subsets of input locality points for the Aa.1 sub-lineage, large southern outbreak and small southern outbreak experiments [file 1472-6785-11-32-S2.PDF]

Table 1. Accuracy metrics for A1.a sub-lineage experiments using random 80%/20% subsets (N to build models=26; N to test models=13). Model designations correspond to figures.

	Model									
**Metric**	**A**	**B**	**C**	**D**	**E**	**F**	**G**	**H**	**I**	**J**
Total Omission	0	0	0	0	0	0	0	0	0	8.3
Average Omission	14.6	15.4	28.3	10.8	7.5	17.5	17.7	21.7	32.5	19.7
Total Commission	22.58	18.69	11.38	24.06	36.26	10.17	10.43	11.73	12.93	15.99
Average Commission	65.2	63.77	48.23	63.88	71.93	59.93	67.49	59.74	56.99	58.23
AUC	0.6584	0.7034	0.6523	0.7527	0.6727	0.7364	0.6862	0.6881	0.5949	0.6255
SE	0.0829	0.0814	0.0864	0.0784	0.0859	0.0828	0.0821	0.0854	0.0866	0.0866
z-score	4.2181	4.4028	3.742	4.866	4.4394	4.3221	4.3662	4.004	3.3957	3.7671

Table 2. Accuracy metrics for small southern outbreak experiments using random 80%/20% subsets (N to build models=26; N to test models=147). Model designations correspond to figures.

	Model									
**Metric**	**A**	**B**	**C**	**D**	**E**	**F**	**G**	**H**	**I**	**J**
Total Omission	0	0.08	9.4	0	35.9	3.1	3.1	0.8	0.8	1.6
Average Omission	40.3	34	31.8	29.5	67	14.6	19	39	20.3	16.8
Total Commission	16.08	6.48	13.06	10.32	6.13	31.77	16.95	6.13	15.99	28.5
Average Commission	46.02	43.09	36.39	50.29	29.08	61.66	51.31	39.37	48.67	59.05
AUC	0.5946	0.6789	0.7154	0.6675	0.5117	0.6323	0.7148	0.6855	0.7003	0.6483
SE	0.0266	0.0264	0.027	0.0264	0.0258	0.0266	0.0259	0.0268	0.026	0.0264
z-score	12.381	14.3437	14.7566	13.9189	12.2182	14.4503	15.1266	13.9527	15.0923	14.6127

Table 3. Accuracy metrics for large southern outbreak experiments using random 85%/15% subsets (N to build models=113; N to test models=145). Model designations correspond to figures.

	Model									
**Metric**	**A**	**B**	**C**	**D**	**E**	**F**	**G**	**H**	**I**	**J**
Total Omission	0	0.7	0	0.7	0	0	0	0.7	0	1.4
Average Omission	20.5	16.5	22.3	21.5	9.6	18.2	16.1	5.1	15.6	16.2
Total Commission	11.7	12.47	12.25	7.49	30.7	17.28	16.2	36.28	15.53	19.59
Average Commission	53.3	50.53	51.51	48.21	60.54	53.14	55.62	63.38	54.05	57.1
AUC	0.7036	0.7361	0.6902	0.7371	0.718	0.7027	0.7138	0.7171	0.7008	0.7
SE	0.0247	0.024	0.0249	0.0241	0.0245	0.0245	0.0245	0.0245	0.0246	0.0246
z-score	15.7135	16.8351	15.3138	16.4132	17.1266	15.9534	16.2323	18.3636	16.1368	16.044
