# Supplementary material for: Ecological Niche Modelling of the Bacillus anthracis A1.a sub-lineage in Kazakhstan
Source: BMC Ecol. 2011 Dec 12;11:32. doi: 10.1186/1472-6785-11-32 (PMC3260114; doi:10.1186/1472-6785-11-32)

Distribution of Soil Variables Used for Outbreak-Soil and Sub-lineage Models

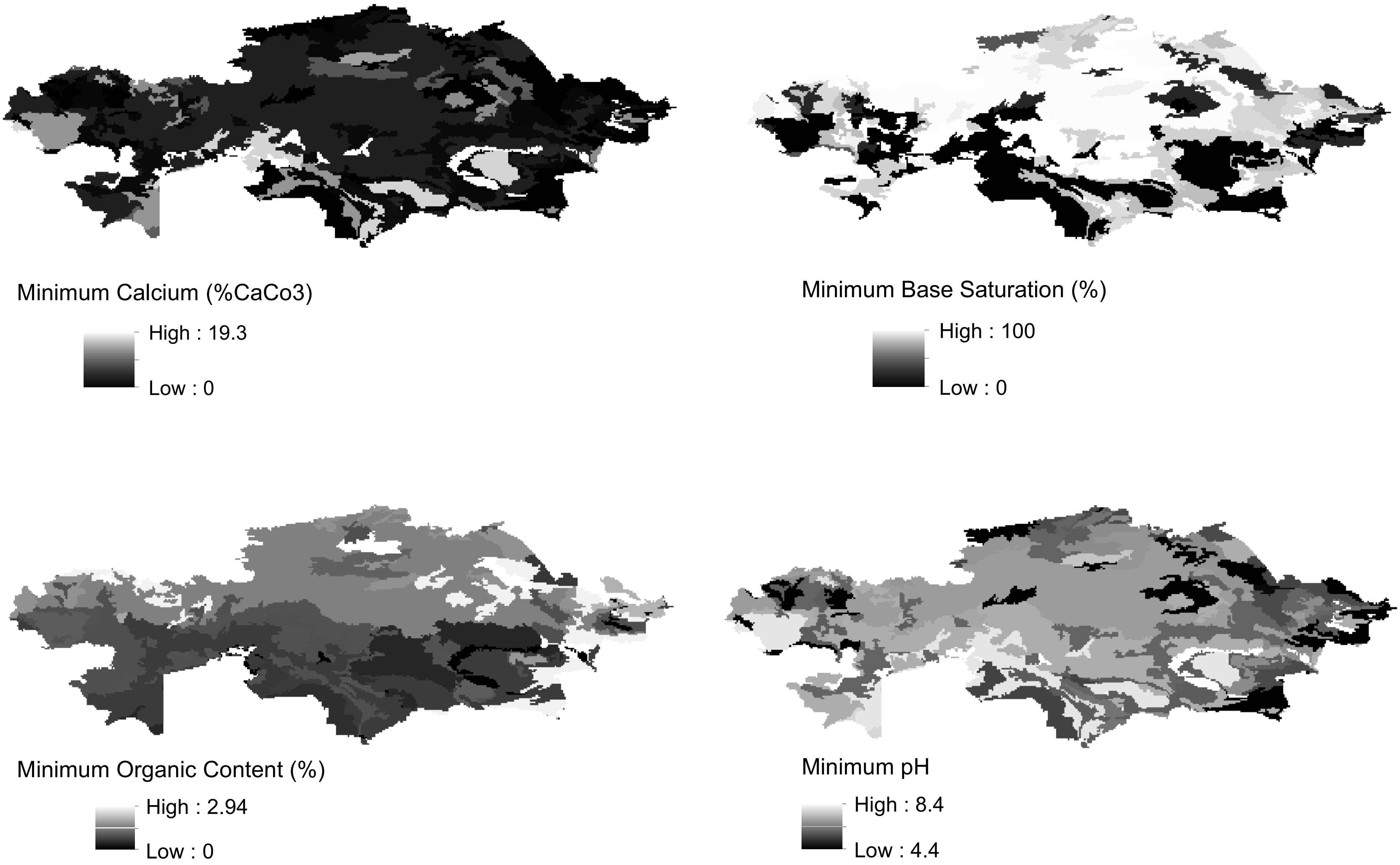

Supplement: Additional file 3 — Soil Variables. Mapped values of the four soil variables (minimum soil pH, minimum soil organic content, minimum soil calcium and minimum soil base saturation). [file 1472-6785-11-32-S3.PDF]
